# Supplementary material for: A Likelihood Approach for Real-Time Calibration of Stochastic Compartmental Epidemic Models
Source: PLoS Comput Biol. 2017 Jan 17;13(1):e1005257. doi: 10.1371/journal.pcbi.1005257 (PMC5240920; doi:10.1371/journal.pcbi.1005257)
Supplement: S1 File — (TAR.GZ) [file pcbi.1005257.s014.tar.gz › HSPH_Online-SI-Revision/EnKF07/n1-severe/n1-severe_EnKF_table-8.pdf]

1	R0	{1.51081, 13.7078}	{2.25808, 6.95349}	{{1.20584, 2.75897}, {3., 13.0053}}	{65.0311, 56.0067}	inf	20.	149.6	{30.1481, 346.913}	651.963	0.71396	newly	8.	134.46	{22., 392.}	1581.5	1.00432	Reff	1.3839	0.514657	{0.114065, 1.04723}	67.2671	ar	656.	1592.83	{1091., 1887.}	143.233
2	R0	{1.73182, 11.3873}	{2.60488, 6.93501}	{{2.15518, 2.54248}, {3., 12.7842}}	{55.1721, 44.042}	inf	10.	210.225	{62.0194, 373.861}	2005.08	1.23305	newly	5.	223.	{52., 505.}	4362.	1.49731	Reff	1.66774	0.848492	{0.237455, 1.58432}	54.5362	ar	653.	1572.46	{1105., 2184.}	142.337
3	R0	{1.50449, 10.3474}	{2.53411, 7.77284}	{{1.64526, 2.63735}, {3., 13.3002}}	{68.4362, 46.5973}	inf	24.	197.655	{61.6502, 355.155}	729.808	0.805212	newly	17.	183.06	{36., 405.}	980.588	0.866325	Reff	1.41873	0.675147	{0.214571, 1.05423}	60.851	ar	651.	1584.34	{1188., 1874.}	143.37
4	R0	{1.63493, 9.1955}	{2.35769, 9.21682}	{{1.62984, 2.20926}, {3., 15.6269}}	{51.6974, 36.7742}	inf	6.	238.856	{70.3991, 375.017}	3882.66	1.48936	newly	4.	256.26	{56., 638.}	6308.	1.601	Reff	1.61368	1.26161	{0.238125, 2.19786}	37.9663	ar	663.	1330.15	{820., 1850.}	102.903
5	R0	{1.53822, 8.05553}	{2.26521, 6.76797}	{{0.978594, 2.32253}, {3., 15.8422}}	{68.7994, 52.2228}	inf	7.	267.318	{64.4192, 500.361}	3718.83	1.48237	newly	6.	341.31	{57., 772.}	5588.5	1.57847	Reff	1.51515	1.01861	{0.359232, 1.77736}	41.1266	ar	621.	1330.05	{713., 1800.}	116.926
6	R0	{1.69374, 17.0793}	{2.20092, 7.79198}	{{0.758035, 3.87761}, {3., 17.1364}}	{54.2905, 56.7287}	inf	15.	147.857	{35.9922, 289.482}	887.8	0.836553	newly	6.	119.56	{23., 288.}	1894.67	1.09115	Reff	1.64801	0.453788	{0.105236, 0.928467}	73.7033	ar	662.	1593.96	{1146., 2113.}	141.103
7	R0	{1.42179, 11.8031}	{2.21172, 7.48224}	{{0.830169, 4.42028}, {3., 14.5482}}	{71.1659, 49.5709}	inf	25.	140.349	{29.6929, 279.578}	467.068	0.608336	newly	16.	120.35	{23., 281.}	657.188	0.688826	Reff	1.34786	0.492039	{0.13122, 1.26212}	67.1284	ar	517.	1588.68	{1187., 2043.}	207.288
8	R0	{1.46683, 6.95875}	{2.36937, 8.97527}	{{1.63539, 2.21275}, {3., 15.5967}}	{67.7941, 51.5875}	inf	9.	239.926	{68.9192, 374.407}	2568.48	1.3271	newly	10.	271.62	{54., 688.}	2621.4	1.25565	Reff	1.44043	1.2777	{0.242077, 2.2399}	39.1144	ar	653.	1314.34	{811., 1831.}	103.342
9	R0	{1.34525, 11.505}	{2.25137, 7.33652}	{{1.18476, 4.27325}, {3., 17.6306}}	{77.9402, 51.7508}	inf	14.	148.145	{21.0996, 303.759}	960.596	0.840467	newly	10.	131.19	{27., 335.}	1216.3	0.90906	Reff	1.2874	0.571535	{0.130504, 1.26487}	61.3253	ar	532.	1610.41	{1104., 2076.}	202.874
10	R0	{1.77958, 14.372}	{2.34553, 7.13741}	{{1.26067, 2.5695}, {3., 15.4364}}	{49.8253, 56.7362}	inf	20.	146.566	{42.6651, 301.895}	634.693	0.733	newly	5.	133.39	{32., 350.}	2568.6	1.23875	Reff	1.68348	0.517718	{0.108282, 1.25941}	71.0585	ar	671.	1592.12	{1030., 2058.}	138.14
11	R0	{1.60334, 19.9548}	{2.38986, 6.63534}	{{1.60235, 3.98726}, {3., 9.0828}}	{55.0927, 66.7482}	inf	26.	55.0613	{4.34493, 107.301}	151.329	0.354328	newly	11.	69.36	{3., 116.}	556.909	0.566068	Reff	1.48149	0.521934	{0.0772744, 0.708523}	67.2935	ar	617.	1805.44	{1633., 2236.}	193.219
12	R0	{1.68623, 7.58585}	{2.30098, 9.44314}	{{0.922814, 2.13956}, {3., 14.1951}}	{53.9501, 52.0196}	inf	4.	199.509	{48.5023, 428.739}	4889.54	1.5646	newly	7.	237.26	{27., 724.}	3291.71	1.29558	Reff	1.67443	1.42572	{0.334728, 2.83267}	41.3381	ar	640.	1123.1	{525., 2038.}	82.6656
13	R0	{1.82689, 18.0022}	{2.24205, 7.33351}	{{1.18371, 4.34373}, {3., 14.7422}}	{47.7609, 60.0279}	inf	23.	128.658	{31.0046, 261.655}	463.723	0.614762	newly	6.	109.38	{24., 211.}	1724.	1.05473	Reff	1.74285	0.463485	{0.130823, 0.933288}	74.2181	ar	673.	1558.6	{1190., 1981.}	131.961
14	R0	{1.75911, 9.16883}	{2.27407, 9.25355}	{{0.841215, 2.13956}, {3., 13.7303}}	{48.5987, 38.7156}	inf	4.	191.631	{51.8015, 412.294}	4692.76	1.56304	newly	3.	245.87	{32., 724.}	8097.67	1.66619	Reff	1.75031	1.43915	{0.191447, 2.4922}	40.2479	ar	661.	1112.87	{508., 1988.}	78.7095
15	R0	{1.46168, 11.3859}	{2.09991, 5.299}	{{0.21239, 1.32505}, {3., 7.38722}}	{80.0035, 59.8017}	inf	25.	101.606	{10.0768, 173.822}	323.169	0.522272	newly	14.	109.19	{15., 251.}	689.357	0.679845	Reff	1.32428	0.382083	{0.0337524, 0.545478}	74.7806	ar	556.	1576.55	{706., 1942.}	187.045
16	R0	{1.53577, 11.7581}	{2.3004, 9.55478}	{{0.807511, 2.14005}, {3., 14.2142}}	{66.9356, 31.7507}	inf	5.	192.717	{42.8638, 413.009}	3756.01	1.44575	newly	2.	221.02	{20., 637.}	10952.	1.79032	Reff	1.52041	1.44497	{0.209029, 2.75435}	44.7307	ar	625.	1135.08	{517., 2038.}	88.9824
17	R0	{1.70698, 9.72924}	{2.28731, 9.48888}	{{0.810461, 2.14055}, {3., 14.2152}}	{53.4125, 34.9616}	inf	8.	190.298	{43.1565, 413.416}	2280.39	1.23696	newly	5.	224.42	{17., 630.}	4390.4	1.40347	Reff	1.68138	1.40601	{0.200474, 2.75726}	42.0958	ar	675.	1117.76	{545., 1894.}	74.6015
18	R0	{1.5973, 14.931}	{2.29736, 9.28695}	{{0.842806, 2.13956}, {3., 13.7208}}	{61.2425, 40.8619}	inf	9.	196.948	{48.3416, 412.455}	2090.27	1.2171	newly	7.	246.31	{31., 732.}	3421.	1.30358	Reff	1.57973	1.44011	{0.334568, 2.83108}	44.1645	ar	632.	1112.48	{547., 1982.}	83.8449
19	R0	{1.87468, 17.004}	{2.56337, 5.67928}	{{2.12963, 0.897687}, {3., 9.5098}}	{39.2299, 66.6003}	inf	36.	59.8008	{13.6671, 105.905}	92.6782	0.233227	newly	15.	103.01	{11., 199.}	599.	0.520919	Reff	1.69659	0.630465	{0.130395, 2.1275}	69.2276	ar	692.	1825.67	{1634., 2486.}	164.814
20	R0	{1.84715, 12.531}	{2.53924, 5.55376}	{{2.12927, 0.606102}, {3., 9.66553}}	{42.3706, 56.7028}	inf	31.	58.5506	{14.4561, 95.2948}	107.873	0.239555	newly	22.	104.29	{13., 237.}	390.318	0.390108	Reff	1.71785	0.60672	{0.126341, 1.63691}	69.8879	ar	661.	1807.83	{1631., 2414.}	174.029
21	R0	{1.75938, 13.5959}	{2.28877, 9.31458}	{{0.82763, 2.14104}, {3., 14.1929}}	{49.3121, 37.5542}	inf	12.	191.885	{39.8865, 428.676}	1501.02	1.05467	newly	10.	227.3	{16., 634.}	2176.4	1.12182	Reff	1.71891	1.41323	{0.206459, 2.03147}	38.7904	ar	620.	1139.51	{545., 2041.}	91.8597
22	R0	{1.63456, 10.7469}	{2.07064, 4.98279}	{{0.190263, 1.86493}, {3., 7.37814}}	{69.4536, 59.1038}	inf	9.	97.1853	{11.7915, 189.602}	983.996	0.826441	newly	9.	99.51	{12., 200.}	1010.56	0.815055	Reff	1.57572	0.377265	{0.0339171, 0.597578}	76.3095	ar	654.	1618.	{1193., 1873.}	150.584
23	R0	{1.62702, 19.1392}	{2.55992, 7.24782}	{{1.7667, 2.64648}, {3., 13.0121}}	{57.4487, 62.1331}	inf	40.	197.18	{65.3756, 367.247}	399.665	0.605069	newly	13.	189.71	{39., 425.}	1362.08	0.995494	Reff	1.4692	0.658707	{0.217837, 1.01423}	62.0005	ar	600.	1578.19	{1093., 2071.}	163.082
24	R0	{1.70466, 12.1202}	{2.5153, 6.00978}	{{2.13771, 0.92069}, {3., 9.60858}}	{53.6931, 52.4004}	inf	32.	71.0055	{17.0948, 146.088}	141.699	0.299787	newly	23.	108.68	{12., 242.}	392.957	0.430202	Reff	1.43191	0.631747	{0.0981656, 2.13712}	68.9117	ar	613.	1822.44	{1622., 2530.}	198.284
25	R0	{1.61735, 11.3169}	{2.25022, 4.48831}	{{0.660437, 1.38831}, {3., 10.8021}}	{69.5118, 61.0381}	inf	25.	56.4314	{5.41391, 42.4977}	174.872	0.393098	newly	15.	70.85	{3., 65.}	404.6	0.499169	Reff	1.53163	0.411695	{0.10112, 0.625054}	75.9045	ar	681.	1796.59	{1635., 1930.}	163.816
26	R0	{1.56977, 12.3035}	{2.26464, 6.77037}	{{0.976, 2.32136}, {3., 15.838}}	{66.6453, 56.8861}	inf	16.	267.409	{64.6336, 500.586}	1571.31	1.12394	newly	8.	341.7	{61., 780.}	4171.5	1.45471	Reff	1.53367	1.01862	{0.362812, 1.77757}	41.5544	ar	594.	1328.22	{690., 1823.}	125.63
27	R0	{1.46317, 5.8298}	{2.55987, 7.27282}	{{2.12008, 2.5252}, {3., 12.7725}}	{82.3823, 49.4609}	inf	8.	227.66	{62.723, 364.991}	2746.01	1.33277	newly	8.	216.43	{64., 542.}	2605.38	1.28451	Reff	1.39294	0.798204	{0.225, 1.53519}	50.7712	ar	586.	1565.71	{1070., 2216.}	167.585
28	R0	{1.70002, 14.9806}	{2.2745, 9.24605}	{{0.840619, 2.14055}, {3., 13.6706}}	{52.512, 41.3129}	inf	4.	191.6	{51.7729, 412.774}	4691.65	1.55501	newly	0.1	244.82	{31., 720.}	244720.	3.12433	Reff	1.68812	1.43652	{0.194114, 2.49511}	41.6734	ar	652.	1112.7	{526., 1988.}	80.0429
29	R0	{1.52729, 16.1014}	{2.56002, 5.42701}	{{2.12921, 0.572983}, {3., 10.1299}}	{70.9941, 66.2948}	inf	40.	59.9228	{12.5052, 102.843}	87.4207	0.256128	newly	22.	107.03	{11., 215.}	406.227	0.424755	Reff	1.40358	0.601224	{0.106, 1.53358}	67.8108	ar	592.	1848.45	{1643., 2485.}	213.262
30	R0	{1.72795, 13.5635}	{2.05834, 5.43897}	{{0.218321, 1.5184}, {3., 7.28985}}	{62.2311, 62.7363}	inf	42.	88.8618	{10.6044, 158.75}	143.362	0.397015	newly	22.	87.	{9., 149.}	316.818	0.530672	Reff	1.5396	0.344518	{0.0381145, 0.529741}	78.5656	ar	668.	1573.57	{838., 1776.}	139.136
31	R0	{1.51591, 18.1544}	{2.21654, 7.33227}	{{1.21225, 4.2185}, {3., 14.5551}}	{63.4691, 60.247}	inf	41.	134.416	{19.6097, 255.646}	247.054	0.447988	newly	22.	112.07	{17., 258.}	421.5	0.552931	Reff	1.4007	0.49013	{0.14116, 0.901735}	67.8946	ar	541.	1580.97	{1211., 1991.}	192.275
32	R0	{1.4282, 10.3534}	{2.63145, 6.94034}	{{2.15548, 2.07644}, {3., 12.9509}}	{86.6297, 42.4127}	inf	4.	210.368	{67.9013, 358.494}	5159.2	1.61812	newly	3.	215.7	{61., 468.}	7090.	1.7095	Reff	1.40535	0.819474	{0.228993, 1.59376}	51.9385	ar	521.	1546.64	{1064., 2192.}	197.685
33	R0	{1.56693, 11.3972}	{2.30065, 9.5505}	{{0.80887, 2.14055}, {3., 14.2246}}	{64.3296, 31.7861}	inf	11.	192.694	{42.9962, 413.255}	1653.87	1.1107	newly	6.	220.46	{17., 631.}	3576.67	1.33214	Reff	1.54029	1.44352	{0.209199, 2.75568}	44.1496	ar	695.	1133.93	{525., 2041.}	73.2619
34	R0	{1.50675, 19.8823}	{2.48687, 6.44552}	{{1.63053, 3.74556}, {3., 9.42128}}	{70.4058, 67.5816}	inf	62.	56.5075	{6.18046, 69.1712}	95.1461	0.528797	newly	30.	63.7	{4., 78.}	176.333	0.433774	Reff	1.22047	0.426581	{0.0198753, 0.657239}	68.6259	ar	587.	1825.24	{1650., 2003.}	210.944
35	R0	{1.72889, 7.76068}	{2.27618, 9.29456}	{{0.82346, 2.14005}, {3., 14.1858}}	{50.6101, 49.7459}	inf	6.	190.73	{37.4399, 413.491}	3080.39	1.34853	newly	7.	227.12	{24., 638.}	3146.57	1.27183	Reff	1.70469	1.40302	{0.202327, 2.04574}	39.5462	ar	650.	1119.28	{499., 1902.}	81.8308
36	R0	{1.62644, 4.12303}	{1.7744, 9.03401}	{{0.375739, 2.00079}, {3., 18.2892}}	{56.9186, 153.424}	inf	5.	73.8595	{15.0041, 151.295}	1384.31	1.03318	newly	11.	179.39	{10., 579.}	1544.45	0.915296	Reff	1.61343	1.44492	{0.327821, 2.68326}	49.5289	ar	652.	644.49	{135., 998.}	49.3543
37	R0	{1.70878, 15.8072}	{2.27015, 4.93877}	{{0.674529, 2.09492}, {3., 10.838}}	{63.8316, 68.7562}	inf	53.	50.2034	{4.3121, 76.5453}	96.3432	0.532002	newly	18.	58.73	{4., 72.}	266.722	0.446087	Reff	1.48835	0.408992	{0.104113, 0.637048}	75.4477	ar	674.	1793.45	{1648., 1859.}	166.091
38	R0	{1.58967, 6.80568}	{2.29578, 9.49106}	{{1.31659, 2.32959}, {3., 15.5547}}	{53.2681, 55.778}	inf	6.	241.356	{76.5195, 375.019}	3924.29	1.49263	newly	10.	238.9	{55., 537.}	2293.8	1.2207	Reff	1.55947	1.31212	{0.266974, 2.23862}	34.8995	ar	610.	1358.24	{752., 1856.}	124.17
39	R0	{1.344, 8.19498}	{2.19859, 7.41931}	{{1.19701, 4.50774}, {3., 15.708}}	{75.496, 41.4179}	inf	10.	131.758	{32.7179, 275.717}	1217.58	0.966158	newly	8.	110.29	{22., 234.}	1279.13	0.942317	Reff	1.2889	0.449121	{0.142166, 0.924467}	67.6379	ar	551.	1589.35	{1189., 2060.}	188.448
40	R0	{1.61509, 7.90825}	{1.79836, 8.82724}	{{0.469736, 2.00079}, {3., 17.18}}	{56.4423, 65.4832}	inf	9.	74.3276	{12.7822, 151.724}	735.573	0.805502	newly	10.	191.1	{8., 647.}	1824.4	0.966688	Reff	1.59894	1.45821	{0.328862, 2.6249}	47.9868	ar	581.	640.49	{143., 974.}	56.7143
41	R0	{1.60017, 5.90605}	{2.26155, 6.82926}	{{0.953773, 2.32136}, {3., 15.7981}}	{63.9497, 62.5964}	inf	8.	268.267	{64.6292, 511.848}	3253.33	1.42446	newly	8.	347.98	{53., 781.}	4249.75	1.45127	Reff	1.56817	1.03102	{0.355602, 1.8114}	42.1268	ar	661.	1354.42	{745., 1893.}	108.475
42	R0	{1.64436, 15.1337}	{2.31545, 6.72759}	{{0.95294, 2.30447}, {3., 14.7097}}	{63.035, 59.5498}	inf	21.	291.498	{70.9284, 581.141}	1290.85	1.03592	newly	9.	353.66	{64., 790.}	3832.22	1.41355	Reff	1.5605	1.03981	{0.377017, 1.73942}	41.8117	ar	640.	1381.52	{731., 2028.}	118.481
43	R0	{1.48917, 11.917}	{2.2689, 4.75956}	{{0.648417, 1.36402}, {3., 10.8481}}	{81.0734, 60.076}	inf	31.	50.3332	{6.80214, 58.1}	116.29	0.329761	newly	19.	57.95	{6., 63.}	233.842	0.361167	Reff	1.38791	0.413297	{0.107411, 0.658471}	73.6763	ar	551.	1778.86	{1641., 1843.}	222.842
44	R0	{1.65577, 7.89317}	{2.3578, 9.21389}	{{1.62923, 2.21197}, {3., 15.5868}}	{50.1258, 43.184}	inf	6.	238.806	{70.1623, 374.746}	3881.88	1.48961	newly	9.	255.23	{57., 624.}	2740.11	1.26628	Reff	1.63093	1.26122	{0.240109, 2.20374}	37.9553	ar	576.	1327.96	{825., 1860.}	131.837
45	R0	{1.6909, 12.3275}	{2.53215, 6.61047}	{{1.73275, 2.18318}, {3., 12.8935}}	{51.678, 52.102}	inf	23.	185.745	{77.9272, 299.361}	712.69	0.812793	newly	14.	190.56	{46., 386.}	1264.43	0.985153	Reff	1.5793	0.634002	{0.20399, 1.06162}	63.8133	ar	675.	1553.58	{1117., 1976.}	130.477
46	R0	{1.62351, 16.0698}	{2.23396, 7.3346}	{{1.18798, 4.33868}, {3., 14.5392}}	{57.7032, 57.3832}	inf	17.	137.513	{32.1765, 280.207}	712.877	0.786439	newly	10.	114.31	{23., 223.}	1047.1	0.893935	Reff	1.55532	0.441462	{0.138056, 0.713234}	72.8021	ar	591.	1590.39	{1209., 2064.}	169.108
47	R0	{1.52372, 4.38599}	{1.7744, 9.03401}	{{0.375739, 2.00079}, {3., 18.2892}}	{61.5737, 142.464}	inf	3.	73.8595	{15.0041, 151.295}	2367.18	1.23284	newly	3.	179.39	{10., 579.}	5888.33	1.39943	Reff	1.51458	1.44492	{0.327821, 2.68326}	52.1083	ar	618.	644.49	{135., 998.}	53.0016
48	R0	{1.44306, 11.788}	{2.29253, 7.18587}	{{1.24567, 2.65888}, {3., 15.2235}}	{70.2103, 52.4486}	inf	30.	150.141	{35.264, 345.01}	405.008	0.553541	newly	21.	132.76	{28., 360.}	537.81	0.630602	Reff	1.32617	0.541539	{0.109042, 1.27156}	64.1956	ar	534.	1632.89	{1050., 2055.}	205.837
49	R0	{1.63624, 11.3893}	{2.3119, 9.13045}	{{1.45882, 2.21331}, {3., 15.5625}}	{50.1464, 39.3327}	inf	10.	250.076	{76.1339, 463.451}	2403.31	1.29347	newly	9.	282.39	{57., 717.}	3041.67	1.30162	Reff	1.60188	1.31859	{0.250983, 2.23993}	35.9825	ar	643.	1353.51	{733., 1875.}	112.465
50	R0	{1.46113, 11.1322}	{2.59037, 7.00464}	{{2.13392, 2.51547}, {3., 12.7912}}	{81.7405, 42.9861}	inf	12.	207.97	{66.6684, 356.645}	1634.2	1.14965	newly	6.	224.12	{57., 526.}	3635.33	1.41687	Reff	1.4246	0.842228	{0.245537, 1.63433}	51.885	ar	501.	1565.44	{1093., 2187.}	213.206
